# Structural and Kinetic Variability of Pathogen Cyclophilins: Functional Diversification and Therapeutic Potential

**DOI:** 10.1021/acsinfecdis.6c00016

**Published:** 2026-04-22

**Authors:** Filippo Favretto, Eva Jiménez-Faraco, Silvia Fruncillo, Nicola Masè, Paola Dominici, Juan A. Hermoso, Alessandra Astegno

**Affiliations:** † Department of Biotechnology, 19051 University of Verona, Strada Le Grazie 15, Verona 37134, Italy; ‡ Department of Crystallography and Structural Biology, Institute of Physical Chemistry Blas Cabrera (IQF), CSIC, Serrano 119, Madrid 28006, Spain

**Keywords:** cyclophilins, pathogens, cyclosporin A, PPIase activity, protein structure, inhibitors

## Abstract

Cyclophilins are a highly conserved family of peptidyl-prolyl *cis–trans* isomerases (PPIases) that play essential roles in protein folding, trafficking, and cellular stress adaptation across all domains of life. While extensively studied in humans, cyclophilins from pathogenic microorganisms have garnered growing interest due to their involvement in virulence, immune evasion, and antimicrobial resistance. Unlike bacteria, which typically encode only one or two isoforms, many protozoan parasites possess expanded cyclophilin repertoires, raising important questions about whether these proteins perform redundant functions or have evolved distinct roles in pathogenesis. Despite their biological relevance, detailed structural and mechanistic studies of microbial cyclophilins remain limited. This review focuses on the structural variability and kinetic diversity of pathogen-derived cyclophilins, emphasizing how differences in three-dimensional architecture, enzymatic kinetics, and ligand-binding properties distinguish these enzymes from their host homologues. It also provides an overview of the therapeutic landscape, highlighting natural product-based pan-selective inhibitors such as cyclosporin A and sanglifehrin A, their nonimmunosuppressive analogues, and the recent development of more selective synthetic scaffolds with improved drug-like properties. Deciphering the structural features, enzymatic behavior, and druggability of microbial cyclophilins is key to unlocking their full therapeutic potential.

## Graphical abstract

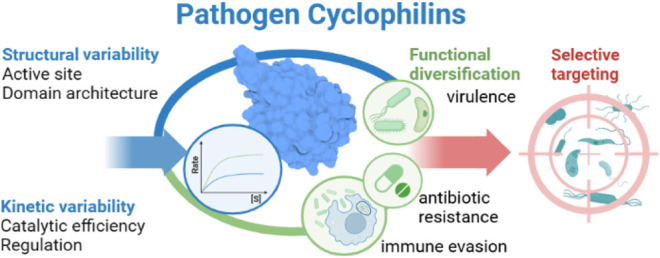

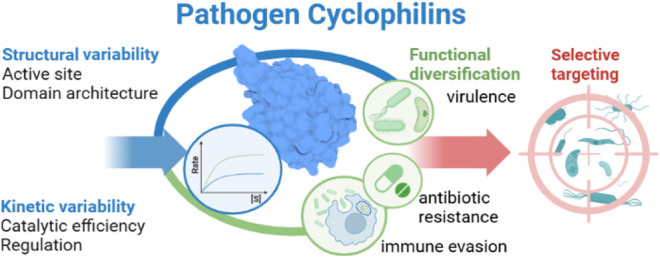

## 1 Introduction

Infectious diseases remain a pressing global health concern, exacerbated by the escalating crisis of antimicrobial resistance. This challenge underscores the urgent need for innovative therapeutic strategies that transcend conventional approaches.

Immunophilins, a superfamily of peptidyl-prolyl *cis–trans* isomerase (PPIase) enzymes, have garnered attention due to their pivotal roles in the virulence, stress tolerance (including oxidative/redox stress responses), adaptation to host environments, and, in several cases, antimicrobial drug resistance of diverse pathogenic microorganisms.^1–5^ This family includes cyclophilins (Cyps), FK506-binding proteins (FKBPs), and parvulins, all of which facilitate the *cis–trans* isomerization of peptidyl-prolyl bonds, a rate-limiting step in protein folding and refolding.^6–9^

Among these, Cyps are particularly noteworthy for their ubiquity and high evolutionary conservation across all domains of life, from prokaryotes to eukaryotes. Cyps are best known for their high-affinity binding to cyclosporin A (CsA), a fungal metabolite with potent immunosuppressive properties.^6,10^ This interaction is central to CsA immunosuppressive effects, wherein the CsA-Cyp complex inhibits calcineurin (Cn), a Ca^2+^/calmodulin dependent serine/threonine phosphatase critical for T-cell activation, thereby suppressing immune responses.^11^ However, Cyps are more than just enzymatic catalysts; they also act as molecular chaperones, assisting in the proper folding, stabilization, and assembly of protein complexes. This dual role positions them as central regulators of proteostasis, particularly under cellular stress, contributing to diverse biological processes, including signal transduction, transcriptional and post-transcriptional regulation, cell cycle control, hormone signaling, vesicular trafficking, and responses to environmental and oxidative stress.^12–17^ In humans, dysregulation of Cyp activity has been linked to multiple pathologies, including cancer, cardiovascular disease, diabetes, and neurodegeneration.^18–20^ In pathogenic microorganisms, Cyps have been directly implicated in virulence, mediating critical interactions with host machinery and contributing to infection outcomes.^1^

One striking feature of Cyp biology is the remarkable diversity in the number and specialization of Cyp family members across different organisms. In bacteria such as *Escherichia coli*, Cyp representation is minimal, typically limited to two proteins, PpiA and PpiB, localized in the periplasm and cytoplasm, respectively.^1^ In contrast, eukaryotes display significant expansion and diversification of Cyp gene families. The human genome encodes 17 genes producing at least 19 proteins via alternative splicing. Plants exhibit even greater complexity, with *Arabidopsis thaliana* harboring 31 genes encoding 48 proteins, *Oryza sativa* (rice) 29 genes yielding 46 proteins, and *Glycine max* (soybean) 62 genes encoding 62 putative Cyp family members. This expansion likely reflects the need for specialized functions across subcellular compartments and adaptive responses to environmental stimuli.^21^ Importantly, protozoan parasites also possess an expanded and diversified set of Cyps. The *Trypanosoma cruzi* Cyp gene family comprises 15 paralogues, *Plasmodium falciparum* 13, and *Toxoplasma gondii* 14.^22–24^ In these pathogens, gene duplication and diversification appear to have driven functional specialization, potentially enabling the parasites to modulate host immune responses, resist environmental stress, or facilitate intracellular survival.

These parasite-specific proteins offer attractive opportunities for selective drug targeting. Besides immunosuppressive properties, CsA also exhibits potent antiparasitic activity. CsA has been shown to block the growth of several protozoan parasites.^25^ This dual role, as both an immunosuppressant and an antiparasitic agent, makes CsA and its derivatives not only therapeutic compounds of interest but also valuable molecular probes. By dissecting their interactions with both host and pathogen Cyps, researchers can elucidate key differences in binding affinity, structure, and function, insights that are fundamental for the rational design of novel Cyp inhibitors.

In this review, we provide an integrated and critical overview of pathogen Cyps, with a primary focus on their structural organization and kinetic properties as a framework to assess their therapeutic potential. We discuss how structural and regulatory features shape ligand recognition and enzymatic activity, and how these properties, together with issues of functional redundancy and specialization within Cyp families, may be leveraged for selective therapeutic targeting. We also review the current landscape of Cyp inhibitors, ranging from natural product-derived pan-selective compounds to emerging nonimmunosuppressive analogues and more selective synthetic scaffolds with improved drug-like properties.

## 2 Roles of Cyps in Pathogenic Microorganisms

Cyps are emerging as key regulators of microbial pathogenesis, integrating protein folding, redox balance, and host signaling to support pathogen survival. Their roles in stress response, immune evasion, and drug resistance underscore their potential as therapeutic targets. Despite growing interest, the specific contributions of individual Cyp family members remain poorly defined, highlighting the need for deeper analysis of their diversity, regulation, and druggability.

### 2.1 Cyps in Protozoan Parasite Virulence and Pathogenesis

Protozoan parasites typically express multiple Cyp proteins, reflecting a diversification of function that supports their complex life cycles and adaptation to host environments. In *T. cruzi*, the causative agent of Chagas disease, this diversity includes at least 15 Cyp members, ranging from organellar proteins to secreted forms. Among them, TcCyp19 is secreted by all developmental stages of the parasite and closely mimics host CypA. The role of the secreted parasite TcCyp19 is the modulation of the intracellular environment within host cells to allow efficient parasite multiplication, exemplifying how Cyps can be co-opted for immune modulation and evasion through molecular mimicry.^26,27^ Meanwhile, another Cyp family member, TcCyp22, localizes to the mitochondrion and plays a key role in the parasite’s response to oxidative stress, functioning analogously to host human CypD. Overexpression of TcCyp22 increases susceptibility to reactive oxygen species by promoting mitochondrial dysfunction and cell death, suggesting that *T. cruzi* Cyps help balance survival and programmed death during oxidative bursts encountered in the host.^28^

A similar functional versatility is evident in *Leishmania* species, where Cyps contribute not only to stress resilience but also to drug tolerance and virulence.^23,29^ Small Cyps from *L. braziliensis* and *L. infantum* have been associated with resistance to antiparasitic drugs, likely through their role in protein folding under chemically stressful conditions.^30^ In *L. donovani*, the larger LdCyp40 protein, which contains a tetratricopeptide repeat (TPR) domain, is essential for full virulence. Parasites lacking this protein show reduced infectivity in macrophages and compensate by upregulating various stress-response pathways, reinforcing the idea that Cyps serve as molecular chaperones that help buffer the parasite against environmental insults such as oxidative stress inside the phagolysosome.^31^ Intriguingly, some *Leishmania* Cyps also elicit protective immunity in animal models, revealing a dual role as both virulence factors and potential vaccine antigens.^32^

In *T. gondii*, Cyps similarly mediate critical aspects of both stress regulation and immune interaction. TgCyp18, a small, secreted protein, binds the host CCR5 chemokine receptor on dendritic cells, triggering cytokine signaling that includes IL-12 induction. This immune activation aids the parasite by fine-tuning host responses, promoting a state that avoids full immune clearance while limiting damaging inflammation.^33–35^ TgCyp18 also shares significant homology with mammalian CypD and carries a mitochondrial targeting sequence; it localizes to the mitochondrial membrane and regulates the permeability transition pore (mPTP). Loss of TgCyp18 impairs cytochrome c release and enhances resistance to oxidative stress-induced cell death, underscoring its vital role in maintaining mitochondrial function and supporting parasite survival under duress.^36^

The link between Cyps and drug resistance becomes particularly apparent in *P. falciparum*, where multiple Cyps are expressed during the parasite’s intraerythrocytic stages. Of particular interest is PfCyp19B, which is upregulated in artemisinin-resistant strains due to a promoter polymorphism. This overexpression appears to confer a survival advantage, potentially by enhancing protein folding capacity or mitigating oxidative damage caused by artemisinin.^37^ PfCyp19B is selectively inhibited by Alisporivir, a nonimmunosuppressive analog of CsA, suggesting a direct mechanistic role in drug resistance and highlighting it as a promising therapeutic target.^38^ Cyp involvement in stress adaptation and transcriptional control is also seen in *T. vaginalis*, where TvCyp1 and TvCyp2 regulate the localization and activity of Myb family transcription factors and protein trafficking. These Cyps translocate Myb proteins from the membrane to the nucleus in response to environmental shifts, facilitating the expression of adhesins and stress-response genes that are essential for host adaptation and pathogenicity.^39–41^

### 2.2 Cyps in Bacterial Pathogenesis

Although bacteria generally encode fewer Cyp proteins than protozoan parasites, typically limited to PpiA and PpiB, these enzymes nonetheless play crucial and versatile roles in supporting bacterial pathogenicity.

The first characterized Cyp in *E. coli*, identified in the early 1990s, demonstrated significant PPIase activity, though with reduced affinity for CsA compared to human CypA.^42^ Both PpiA and PpiB from *E. coli* contribute to essential cellular processes, including protein folding and complex assembly. The cytoplasmic PpiB influences motility and biofilm dynamics: its deletion results in hypermotility and increased biofilm formation, indicating that it helps regulate the correct folding or function of adhesins and flagellar proteins.^43,44^ This control mechanism ensures that motility and surface attachment remain finely tuned, preventing disordered behavior that could compromise infection strategies. Similarly, in *B. pseudomallei*, the loss of BpPpiB impairs biofilm formation and reduces motility, reinforcing the idea that bacterial Cyps help maintain structural and functional integrity under environmental stress.^45^

In Gram-positive pathogens like *Staphylococcus aureus*, Cyps are key to virulence through their influence on protein secretion and stability. The intracellular SaPpiB facilitates the function of secreted toxins and immune modulators. For instance, it is required for the proper folding and activity of Staphylococcal nuclease (Nuc), a key factor in immune evasion, and its absence leads to a marked decrease in virulence determinants such as α-toxin and phenol-soluble modulins.^46,47^ These reductions compromise the bacterium ability to survive inside macrophages and epithelial cells, as well as its virulence in animal models. Interestingly, SaPpiB influence on toxin production appears to be independent of its isomerase activity and is likely related to its interaction with the protein secretion (Sec) translocation machinery, suggesting a broader role in managing protein export under stress.^48^

Unlike PpiB, PpiA is not present in all bacteria, suggesting a less essential role for bacterial viability. *Streptococcus pneumoniae* expresses a surface-anchored Cyp, SlrA (also known as PpiA), which acts at the cell wall interface to assist the folding of exported and membrane-associated proteins. Disruption of SlrA significantly reduces the pneumococcus ability to adhere to airway cells and colonize the respiratory tract, highlighting its importance in the early stages of infection and in resisting phagocytic clearance.^49^ In *Klebsiella pneumoniae*, PpiA is among the genes highly expressed during infection,^50^ while in *E. coli* and *Pseudomonas aeruginosa*, its deletion reduces motility and increases antibiotic susceptibility.^51–53^ In *Acinetobacter baumannii*, overexpression of PpiA correlates with resistance to cefiderocol, linking Cyp expression directly to antimicrobial defense mechanisms.^54^

Even with their limited number, bacterial Cyps often have overlapping or compensatory roles alongside other folding catalysts. For instance, *Legionella pneumophila* encodes both PpiB (cytosolic) and a distinct FKBP-type PPIase called Mip on its surface; these two PPIases together modulate *Legionella* motility, stress tolerance, and intracellular infection capacity.^55^ PpiB in *L. pneumophila* promotes growth at lower temperatures, whereas Mip facilitates the early stages of invading host cells. Double mutants lacking both PpiB and Mip show additive defects in gliding motility on surfaces and synergistic attenuation in infecting amoebae and human macrophages. This suggests each PPIase aids different aspects of *L. pneumophila* fitness yet both converge to support environmental survival and virulence in a complementary fashion.^56^ In *Mycobacterium tuberculosis*, both PpiA and PpiB are deeply integrated into the bacterium pathophysiology. PpiB is required for robust biofilm formation and antibiotic tolerance, while both Cyps actively modulate host immune responses.^57^ Recombinant *M. tuberculosis* PpiA stimulates proinflammatory cytokines such as TNF-α and IL-6, while PpiB triggers an anti-inflammatory response marked by IL-10 induction, promoting bacterial survival in macrophages.^58^

Across both protozoan parasites and bacteria, Cyps consistently emerge as key contributors to pathogenic success, with several recurring themes. They act as molecular mimics and modulators of host–pathogen signaling, enhance defenses against oxidative stress, and contribute to drug resistance mechanisms that undermine chemotherapy. A unifying feature is their role in helping pathogens withstand the stressful conditions imposed by the host. These findings suggest that Cyps are not only central to how pathogens thrive under hostile conditions but could also represent “Achilles’ heels” that might be exploited therapeutically across different taxa.

## 3 Structural Variability of Pathogen-Derived Cyps

This section provides an overview of the principal structural features of Cyps, with a focus on pathogen-derived members. The overall architecture of the cyclophilin-like domain (CLD), a conserved structural module shared across the Cyp family and responsible for enclosing the catalytic site, is first described. The structural diversity of microbial Cyps is then examined, highlighting sequence variations and distinctive structural elements or accessory domains that distinguish them from their canonical human counterparts. Particular attention is devoted to protozoan Cyps, using those from *T. cruzi* and *T. gondii* as representative models, as these organisms encode expanded and diversified Cyp repertoires.

### 3.1 Structural Overview of the Cyp-Like Domain

The defining structural hallmark of Cyps is the Cyp-like domain (CLD), a conserved catalytic module of approximately 109 amino acids. This domain adopts a compact, stable fold characterized by an eight-stranded antiparallel β-barrel flanked by two α-helices (Figure 1A). Within the CLD lies a shallow active site composed of conserved hydrophilic and hydrophobic residues essential for catalyzing *cis–trans* isomerization of peptidyl-prolyl bonds, known also as S1′ pocket (Figure 1B).

**Figure 1:**
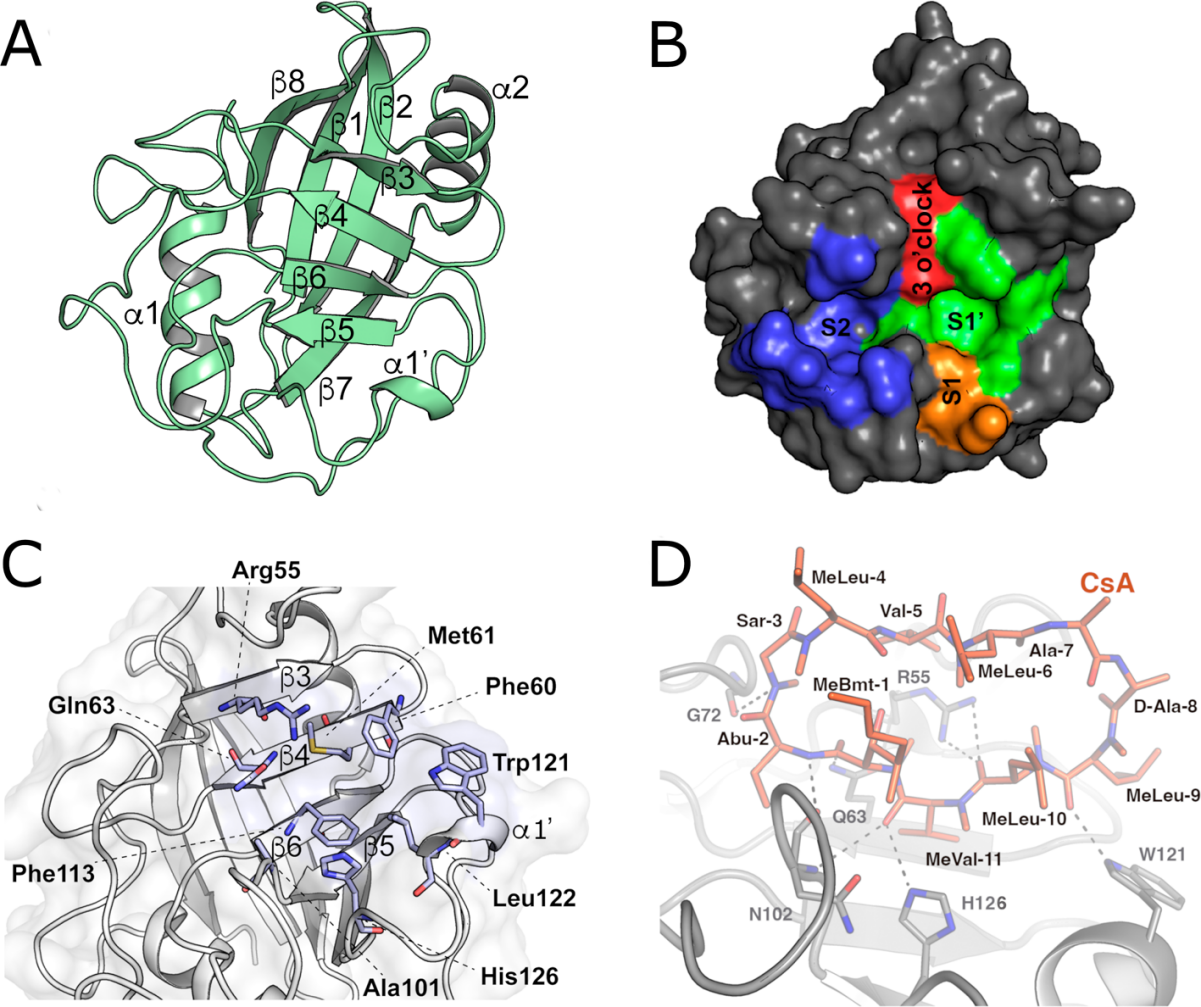
3D fold of the CLD and definition of the critical active site residues. (A) Overall 3D structure of the CLD. Conserved secondary structural elements are labeled on the structure, which was generated using human CypA (PDB entry 1CWA). (B) Surface representation of the human CypA and its sites. Pockets S1′, S2, S1, and “three o’clock” are highlighted in green, blue, orange, and red, respectively. (C) Close-up view of the human CypA binding site (PDB: 1CWA). Residues critical for PPIase activity and for forming the CsA-binding surface are labeled. (D) Interaction pattern between human CypA and CsA (extracted from PDB: 1M63). CypA is represented in gray cartoon and CsA is depicted in orange sticks. Polar interactions are shown with dashed lines.

Numerous Cyp structures have been solved (Table 1), revealing that catalytically active proteins containing a single CLD are typically monomeric, with a few exceptions.^59,60^ The first detailed structural insight into Cyp function came from studies on human CypA, a ubiquitous housekeeping protein and the first of the 19 human Cyps to be identified. Its three-dimensional structure was independently solved in both the apo state and in complex with peptide substrates (Table 1).^61–66^ The crystal structure of CypA in complex with substrate succinyl-Ala-Ala-Pro-Phe-nitroaniline (AAPF) shows that AAPF binds to the hydrophobic pocket on the surface of the CypA barrel.^62,67^ Key interactions involve the invariant catalytic residue Arg55 and a conserved set of hydrophobic, aromatic, and polar residues (Phe60, Met61, Gln63, Gly72, Ala101, Phe113, Trp121, Leu122, and His126) crucial for substrate binding and catalysis (Figure 1C). Gln63 is located near the catalytic arginine and is an important residue, acting as a bridging element in a hydrogen bond network that stabilizes the proper orientation of nearby residues Arg55 and Gln111. Thus, Arg55 is well-oriented to the substrate (Figure 1C). As expected for an enzyme catalyzing *cis–trans* isomerization, both conformers of a proline-containing peptide are presumed to bind the active site. However, most available crystal structures of Cyp-peptide complexes capture the substrate in the *cis* conformation, possibly reflecting a preferred binding mode. High-resolution structures of human CypA bound to CsA (Table 1) reveal that CsA binds to the same active-site groove that accommodates peptide substrates (Figure 1D), with the cyclic undecapeptide ring interacting via both hydrophobic contacts and polar bonds. Key pocket residues (human CypA numbering) including Ile57, Phe60, Met61, Ala101, Phe113 and Leu122 form a hydrophobic cavity that accommodates the MeLeu-Pro segment of CsA (analogous to a peptidyl-proline substrate). However, the stabilization of CsA also involves a network of hydrogen bonds with CypA, mediated by residues Arg55, Gln63, Gly72, Asn102, Trp121 and His126 (Figure 1D). Overall, CsA binding causes no major conformational change in CypA; the β-barrel fold and side-chain positions are essentially unchanged from the apo enzyme. Notably, multiple C–H···O hydrogen bonds (from CsA methyl groups to protein carbonyls) have been observed in these complexes, a feature that contributes to tight binding.^68^

**Table 1:** Selected Solved Cyp Structures from Humans and Pathogens

**Organism**	**UniProt code**	**Protein**	**Ligand**	**PDB Code**	**Refs**
*H. sapiens*	P62937	CypA		2CPL	^65^
*H. sapiens*	P62937	CypA	–Ala-Pro dipeptide	2CYH	^62^
			–Ser-Pro dipeptide	3CYH	
			–His-Pro dipeptide	4CYH	
			–Gly-Pro dipeptide	5CYH	
*H. sapiens*	P62937	CypA	Ala-Ala-Pro-Phe tetrapeptide	1RMH	^67^
*H. sapiens*	P62937	CypA	CsA	1CWA	^63^
*H. sapiens*	P62937	CypA	CsA and Cn	1M63	^73^
*H. sapiens*	P62937	CypA	Voclosporin	3ODI	^74^
*H. sapiens*	P62937	CypA	Alisporivir	5HSV	^75^
*H. sapiens*	P62937	CypA	Sanglifehrin	1YND	^76^
*H. sapiens*	P23284	CypB	[D-Ser(O-cholinyl ester)]-CsA	1CYN	^77^
*H. sapiens*	Q13427	CypG (only CLD)		2WFI	^78^
*H. sapiens*	O43447	CypH		1QOI	^79^
*H. sapiens*	O43447	CypH	U4/U6 snRNP peptide	1MZW	^80^
*H. sapiens*	Q9H2H8	CypJ		1XYH	^81^
*B. malayi*	Q27450	BmCyp1		1A58	^82^
*S. mansoni*	Q26548	SmCypA		2CMT	^83^
*C. parvum*	A3FQA7	CpCyp		2PLU	*To be published*
*C. parvum*	A3FQA7	CpCyp	CsA	2POY	*To be published*
*L. donovani*	Q9U9R3	LdCypA		2HAQ	^84^
*L. donovani*	Q9U9R3	LdCypA	CsA	3EOV	^85^
*L. major*	Q4QBH1	LmCyp11		2HQJ	*To be published*
*P. falciparum*	Q25756	PfCyp19A	CsA	1QNG	^86^
*P. yoelii*	Q7RSH5	PyCyp		1Z81	^87^
*P. yoelii*	Q7RRM6	PyCyp2		2B71	^87^
*T. gondii*	A0A125YL73	TgCyp23	CsA	8B58	^88^
*T. gondii*	A0A125YL73	TgCyp23	–NIM811	8R7S	^89^
			–Alisporivir	8R7T	
			–dihydroCsA	8R7U	
*T. gondii*	A0A125YVH7	TgCyp64 (only CLD)		3BKP	*To be published*
*T. gondii*	A0A125YUW2	TgCyp69 (only CLD)	CsA	3BO7	*To be published*
*T. vaginalis*	A2DT06	TvCyp1 (Cyp19)		5YB9	^60^
*T. vaginalis*	A2DLL4	TvCyp2 (Cyp19.9)		6LXO	^41^
*T. cruzi*	Q4DPB9	TcCypB		1XO7	*To be published*
*T. cruzi*	Q4DPB9	TcCypB	CsA	1XQ7	*To be published*
*B. pseudomallei*	Q63SS5	BpPpiB		3S6M	*To be published*
*E. coli*	P0AFL3	EcPpiA		1J2A	^90^
*E. coli*	P23869	EcPpiB		2NUL	^91^
*S. pyogenes*	Q9A156	SpPpiB		7L6Y	^92^
*M. tuberculosis*	P9WHW3	MtPpiA		1W74	^93^
*S. pneumoniae*	Q8DQG5	SpPpiA		7L6Z	^92^

Beyond the canonical proline-binding pocket, Cyps also feature an auxiliary S2 pocket, whose accessibility is governed by so-called “gatekeeper” residues (Figure 1B). In CypA, these include Thr73, Glu81, Lys82, Ala103, Thr107, Ser110, and Gln111.^69^ These gatekeeper residues show chemical diversity across Cyps family members, making the exosite S2 pocket an attractive target for designing protein-specific inhibitors with enhanced affinity and selectivity. Recently, macrocycle-based inhibitors were identified from a DNA-templated library that bind CypD with high affinity and subtype selectivity. These compounds engage both the active site and the adjacent, nonconserved S2 pocket, enabling up to >10,000-fold selectivity over other Cyps.^70^ Exploiting the S2 pocket further allowed the development of the first selective inhibitor for CypE, which forms a reversible covalent bond with a lysine unique to its S2 pocket. This strategy demonstrates how targeting the variable S2 pocket can yield potent, isoform-selective Cyp inhibitors.^70^ In addition to the S1′ and the S2 pockets, Cyps also possess an S1 pocket, oriented perpendicular to S1′, and a “three o’clock” pocket, which is positioned perpendicular to the S1′-S2 pocket axis and opposite to the S1 pocket (Figure 1B). Both S1 pocket and the “three o’clock” pocket represent sites of potential structural diversity among Cyps that could be exploited for the development of selective inhibitors.^71,72^

### 3.2 Distinctive Structural Features of Pathogen Cyps

To enable in-depth analysis of the variability of residues involved in substrate recognition across different pathogenic species, multiple sequence alignments were performed using human CypA as the reference. Table 2 summarizes this analysis, highlighting the most significant changes within the active site of Cyps from selected bacteria and protozoa. While highly conserved overall, pathogen-derived Cyps also exhibit sequence variability.

**Table 2:** Variability of the Residues Involved in the Active Site across Different Species^a^

**Organism and protein**	**UniProt code**	**Residues**								
****H. sapiens****		55	60	**61**	63	101	113	**121**	122	126
hCypA	P62937	Arg	Phe	Met	Gln	Ala	Phe	Trp	Leu	His
****E. coli****										
EcPpiA	P0AFL3	Arg	Phe	Met	Gln	Ala	Phe	**Phe**	Leu	**Gln**
EcPpiB	P23869	Arg	Phe	Met	Gln	**Arg**	Phe	**Phe**	Leu	**Tyr**
****K. pneumoniae****										
KpPpiA	W1DNJ8	Arg	Phe	Met	Gln	**Arg**	Phe	**Phe**	Leu	**Gln**
KpPpiB	W1DRQ1	Arg	Phe	Met	Gln	**Arg**	Phe	**Phe**	Leu	**Tyr**
****B. pseudomallei****										
BpPpiB	Q63SS5	Arg	Phe	Met	Gln	**Arg**	Phe	**Phe**	Leu	**Tyr**
****S. pneumoniae****										
SpPpiA	Q8DQG5	Arg	Phe	Met	Gln	Ala	Phe	**Ser**	Leu	His
****M. tuberculosis****										
MtPpiA	P9WHW3	Arg	Phe	Met	Gln	Ala	Phe	**His**	Leu	His
****L. major****										
LmCyp19	O02614	Arg	Phe	Met	Gln	Ala	Phe	Trp	Leu	His
****P. falciparum****										
PfCyp19A	Q25756	Arg	Phe	Met	Gln	Ala	Phe	Trp	Leu	His
PfCyp19B	Q8IIK8	Arg	Phe	Met	Gln	Ala	Phe	Trp	Leu	His
****T. vaginalis****										
TvCyp19.9	A2DLL4	Arg	Phe	Met	Gln	Ala	Phe	Trp	Leu	His
****T. gondii* strain Me49***										
TgCyp18	S8F7V1	Arg	Phe	Met	Gln	Ala	Phe	Trp	Leu	His
TgCyp18.4	A0A125YZ79	Arg	Phe	**Ala**	Gln	Ala	Phe	**His**	Leu	**Tyr**
TgCyp21	A0A125YV51	Arg	Phe	**Cys**	Gln	Ala	Phe	**Phe**	Leu	His
TgCyp23	A0A125YL73	Arg	Phe	Met	Gln	Ala	Phe	Trp	Leu	His
TgCyp26	A0A125YLU4	Arg	Phe	Met	Gln	Ala	Phe	Trp	Leu	His
TgCyp32	S8FB56	Arg	Phe	Met	Gln	Ala	Phe	Trp	Leu	His
TgCyp35	A0A125YQ35	**Leu**	**Val**	**Asn**	**Thr**	**Met**	**Ile**	**Pro**	Leu	**Tyr**
TgCyp38	S8F5I7	Arg	Phe	Met	Gln	Ala	Phe	Trp	Leu	His
TgFCB57^b^	S8F548	Arg	Phe	Met	Gln	Ala	Phe	**His**	Leu	His
TgCyp64	A0A125YVH7	Arg	Phe	**Ile**	Gln	Ala	Phe	**Val**	Leu	**Tyr**
TgCyp66.21	A0A125YII8	Arg	**Val**	**Leu**	Gln	Ala	**Tyr**	**Ala**	Leu	His
TgCyp66.25	S8GFQ1	Arg	Phe	Met	Gln	Ala	**Tyr**	**Phe**	Leu	**Leu**
TgCyp69	A0A125YUW2	Arg	Phe	Met	Gln	Ala	Phe	**His**	Leu	His
TgCyp86	S8FD30	Arg	Phe	Met	Gln	Ala	Phe	Trp	Leu	His
****T. cruzi* strain CL Brener***										
TcCyp19 (TcCypA)	Q4E4L9	Arg	Phe	Met	Gln	Ala	Phe	Trp	Leu	His
TcCypB	Q4DPB9	Arg	Phe	Met	Gln	Ala	Phe	Trp	Leu	His
TcCyp20	Q4DC03	Arg	**Ala**	**Phe**	**Leu**	Ala	Phe	**Ser**	Leu	His
TcCyp21	Q4DNC9	Arg	Phe	Met	Gln	Ala	Phe	Trp	Leu	His
TcCyp22	Q4DI85	Arg	**Gly**	**Leu**	**Val**	**Cys**	Phe	**Ser**	Leu	His
TcCyp24	Q4CXV1/Q4D7C3	Arg	Phe	Met	Gln	Ala	Phe	Trp	Leu	His
TcCyp25	Q4DFL3	Arg	Phe	**Ile**	Gln	Ala	Phe	**His**	Leu	**Tyr**
TcCyp26.2	Q4DJE5	Arg	**Ser**	**Leu**	**Arg**	**Cys**	Phe	**Ser**	Leu	His
TcCyp28	Q4CX88	Arg	Phe	Met	Gln	Ala	Phe	Trp	Leu	His
TcCyp26	Q4DU72	**Asp**	**Gln**	**Tyr**	**Ile**	**Gln**	**Arg**	**Lys**	**Met**	**Ala**
TcCyp30	Q4DNS3	Arg	**Tyr**	**Leu**	Gln	Ala	Phe	**His**	Leu	**Cys**
TcCyp35	Q4DM35	Arg	**Ala**	**Trp**	**Met**	**Cys**	**Tyr**	Trp	**Met**	**Tyr**
TcCyp29	Q4DQI8	Arg	**Arg**	**Leu**	**Leu**	**Val**	**Gly**	**Ser**	Leu	**Gln**
TcCyp40	Q4E4G0	Arg	Phe	Met	Gln	Ala	Phe	**His**	Leu	His
TcCyp103	Q4D1M5	Arg	Phe	Met	Gln	Ala	Phe	Trp	Leu	His

a *Residue numbering refers to hCypA. Non-conserved residues are highlighted in bold* *.*

b *TgFCB57 is a peculiar PPIase of* *T. gondii* *that, in addition to the canonical CLD, also harbors an FKBP domain.*

In bacteria, PpiB from *E. coli*, despite preserving the general architecture of human CypA, shows structural differences, particularly in loops L_α1−β3_, L_β4−β5_, L_β6−β7_, and L_α2−β8_.^91,94^ These regions contain amino acid substitutions or insertions that lie along the hydrophobic cleft of the CsA-binding core, potentially preventing CsA entry into the protein hydrophobic groove. Furthermore, in bacterial Cyps, Trp121 is often substituted with phenylalanine (Phe), and His126 with tyrosine (Tyr) (Table 2). In protozoa Cyps, the catalytic arginine is generally conserved, with the exception of TgCyp35 in *T. gondii* and TcCyp26 in *T. cruzi*, which lack residues involved in substrate recognition (Table 2). Gln63 is also highly conserved across nearly all analyzed sequences, suggesting a key role in substrate stabilization, likely through hydrogen-bonding interactions with the catalytic arginine that helps maintain its proper orientation within the catalytic site (Figure 1B). In contrast, residues Met61 and Trp121 (numbered according to human CypA) show high variability. In *T. gondii*, eight Cyps exhibit substitutions at position Trp121, and five at Met61. In *T. cruzi*, the residues exhibiting the greatest variability are Phe60, Met61, Trp121, and His126, with approximately half of the proteins harboring substitutions at one or more of these positions. Collectively, these four residues may represent promising targets for the rational design of selective inhibitors. Specifically, these mutations dramatically reduce CsA binding affinity, from the nanomolar to the micromolar range, without fully compromising PPIase activity.^95^

In *T. gondii*, TgCyp18.4 harbors substitutions at key CsA-contact positions. Notably, His111 replaces the conserved tryptophan (Trp121 in human CypA) that normally forms a hydrogen-bond with CsA. In addition, Tyr116 (instead of His126 in human CypA) and Gln92 (instead of Ala103 in human CypA) alter the hydrogen-bond geometry, weakening the network of polar interactions and accounting for the markedly reduced CsA affinity of TgCyp18.4. Mutation of His111 to Trp restores high-affinity binding.^88^ Overall, these observations indicate that catalytic efficiency can be maintained despite alterations in residues involved in ligand recognition, highlighting a degree of structural plasticity tolerated in microbial enzymes.

Beyond the catalytic domain, many pathogen-derived Cyps display additional structural complexity. Cyps are broadly classified as either single-domain (containing only the CLD) or multidomain, integrating other functional elements.^9^ For example, human CypH contains a small insertion in a loop that creates a second, highly specific protein–protein interaction site distinct from its catalytic pocket, enabling stable association with proteins like the 60K component of the U4/U6 snRNP complex.^80^ Larger Cyps, such as Cyp40, are modular, possessing multiple PPIase and/or protein–protein interaction domains, including tetratricopeptide repeats (TPRs) necessary for binding to Hsp90. TPRs are structural motifs frequently found in protein–protein interactions and consist of about 34 amino acids arranged in two parallel α-helices.^96^

Many pathogenic species possess multidomain Cyps, which significantly expand their functional repertoire and represent structurally attractive targets.^4^ A ubiquitous class is the WD40-repeat Cyps, which fuse the CLD to a WD40 domain.^4,24^ The WD40 module (tandem ∼ 40 aa Trp/Asp repeats) varies among species (e.g., only ∼145 aa in *P. falciparum* versus ∼320 aa in *T. gondii* *)*. WD40 Cyps are typically nuclear and are thought to engage in RNA splicing or chromatin interactions. Another conserved architecture is the RING-Cyp fusion. In this PPIL2-like subfamily, an N-terminal Cys/His-rich RING (zinc-finger) motif (also known as a U-box) precedes the CLD.^24,97^ For example, *Toxoplasma* TgCyp69 carries a U-box domain followed by a CLD. Strikingly, this RING-Cyp class is absent in *P. falciparum*, indicating lineage-specific loss. U-box domains typically confer E3 ubiquitin-ligase activity, suggesting that Cyps may regulate parasite protein ubiquitination or degradation. These RING-Cyp proteins often possess nuclear localization signals, pointing to nuclear ubiquitin-proteasome or signaling roles (Figure 2).

**Figure 2:**
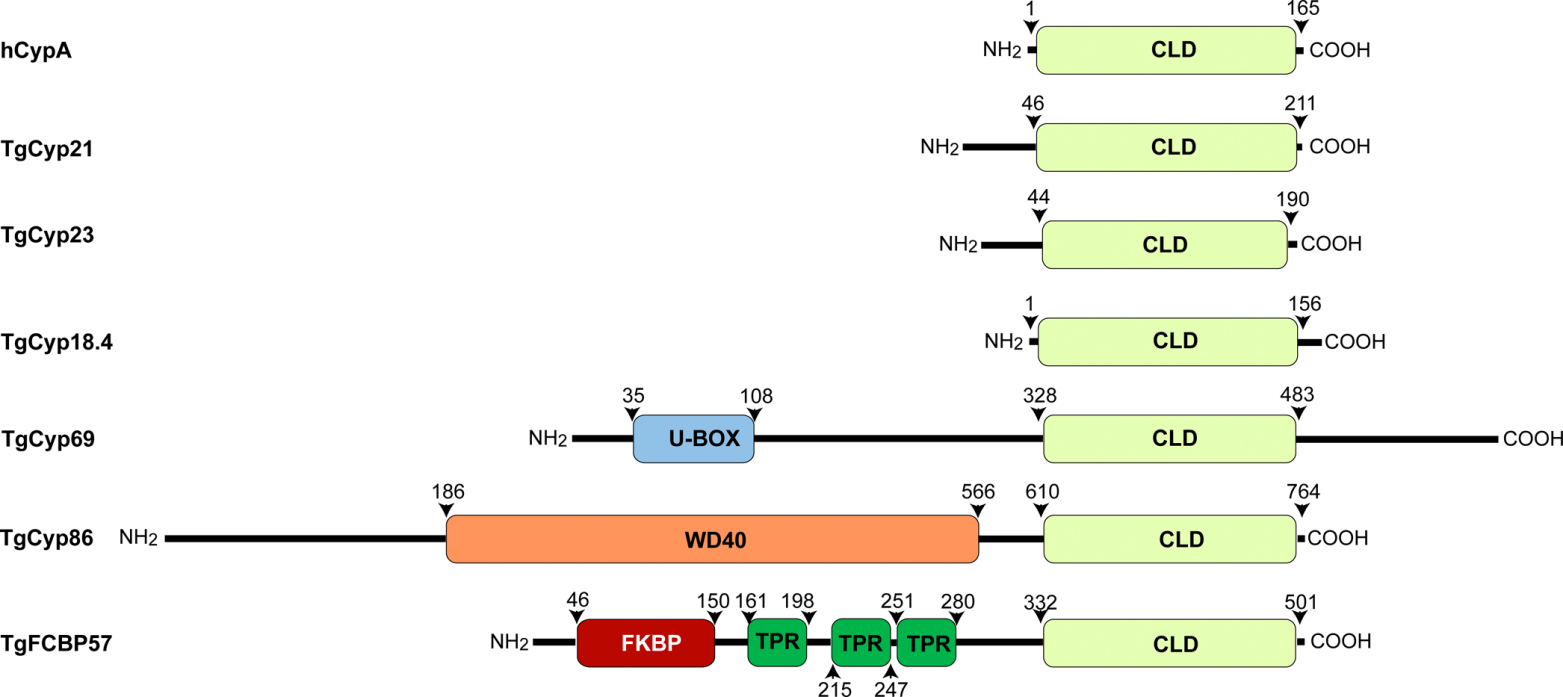
Domain organization of *T. gondii* Cyps. Representative members of the Cyp family in *T. gondii* are shown, with their domain architecture annotated. Domain types and positions are indicated along with the corresponding amino acid numbers.

Beyond these subfamilies, apicomplexan Cyps exhibit additional unique domain features. *Toxoplasma* encodes RRM (RNA-recognition motif)-containing Cyps, hinting at roles in mRNA metabolism or spliceosome-associated functions. Furthermore, a novel group of dual Cyps has been described in *Toxoplasma*, containing both an N-terminal FKBP (FK506-binding protein) domain and a C-terminal CLD, joined by three intervening TPR repeats (Figure 2). These FKBP-Cyp chimeras previously identified only in archaea or eubacteria and absent from *Plasmodium*, suggest a case of functional convergence, possibly enhancing substrate versatility or complex formation in parasitic organisms.

Notably, many pathogen-derived Cyps also contain transmembrane helix (TMH) segments and N-terminal or C-terminal extensions that may serve as signal peptides or aid in cellular localization.^4^ For example, putative mitochondrial Cyps have been identified in several human parasitic species, including *P. falciparum* and *T. gondii*. These Cyps carry large N-terminal extensions compatible with mitochondrial localization signals. In *T. vaginalis*, TvCyp2 (TvCyp19.9) features an N-terminal segment with a strong propensity to form α-helical structures, supporting its role in enzyme localization and association with biological membranes. The crystal structure of TvCyp2 confirms the canonical CLD fold yet also shows that the N-terminal extension can occupy the active site of adjacent molecules within the crystal lattice, raising intriguing questions about possible regulatory mechanisms mediated by autoinhibitory interactions.^41^ Similarly, TgCyp23 from *T. gondii* possesses a 39-amino acid N-terminal extension with helical propensity and a possible role in subcellular targeting.^88^

To investigate the structural differences between protozoan Cyps and human CypA, we superimposed the structures of *T. gondii* and *T. cruzi* Cyps (Figure S1) onto human CypA. Some of them have already been solved by X-ray crystallography (e.g., TgCyp23 and TcCypB) and for the remaining ones we used AlphaFold^98–100^ to predict their structures. Structural analysis (Figure 3) revealed six regions of significant variability. The highest structural divergence among *T. gondii* and *T. cruzi* Cyps was observed in the N- and C-terminal regions, as well as in the loops L_β1−β2_, L_α1−β3_, L_β5−β6_, and L_α2−β8_ (Table S1, S2, Figure 3, and Figure S1). A representative example is TcCyp26 from *T. cruzi* (Figure 3C), which displays longer L_β1−β2_, L_β5−β6_, and L_α2−β8_ loops compared to human CypA. Notably, the elongation of loop L_β5−β6_ may have functional implications, as it is positioned adjacent to the active site and could potentially interfere with catalytic activity. Consistent with this expanded architecture, the N-terminal region of TcCyp26 also contains 24 additional residues. Another distinctive feature is the extension of the L_α1−β3_ loop, which appears to be conserved among most of the *T. cruzi* Cyps (Table S2). In this Cyp, the extra region is composed of two antiparallel β-strands linked by a tight turn, forming a characteristic β-hairpin fold (highlighted by a yellow arrow in Figure 3C). Similarly, TgCyp18.4 from *T. gondii* exhibits notable structural differences compared to human CypA (Table S1, Figure 3D). In contrast to TcCyp26, TgCyp18.4 has shorter L_β1−β2_ and L_α1−β3_ loops, along with an additional C-terminal α-helix that is absent in human CypA. However, the L_α2−β8_ loop is longer than in human CypA and adopts a β-hairpin fold composed of two antiparallel β-strands linked by a tight turn (β-hairpin folding).

**Figure 3:**
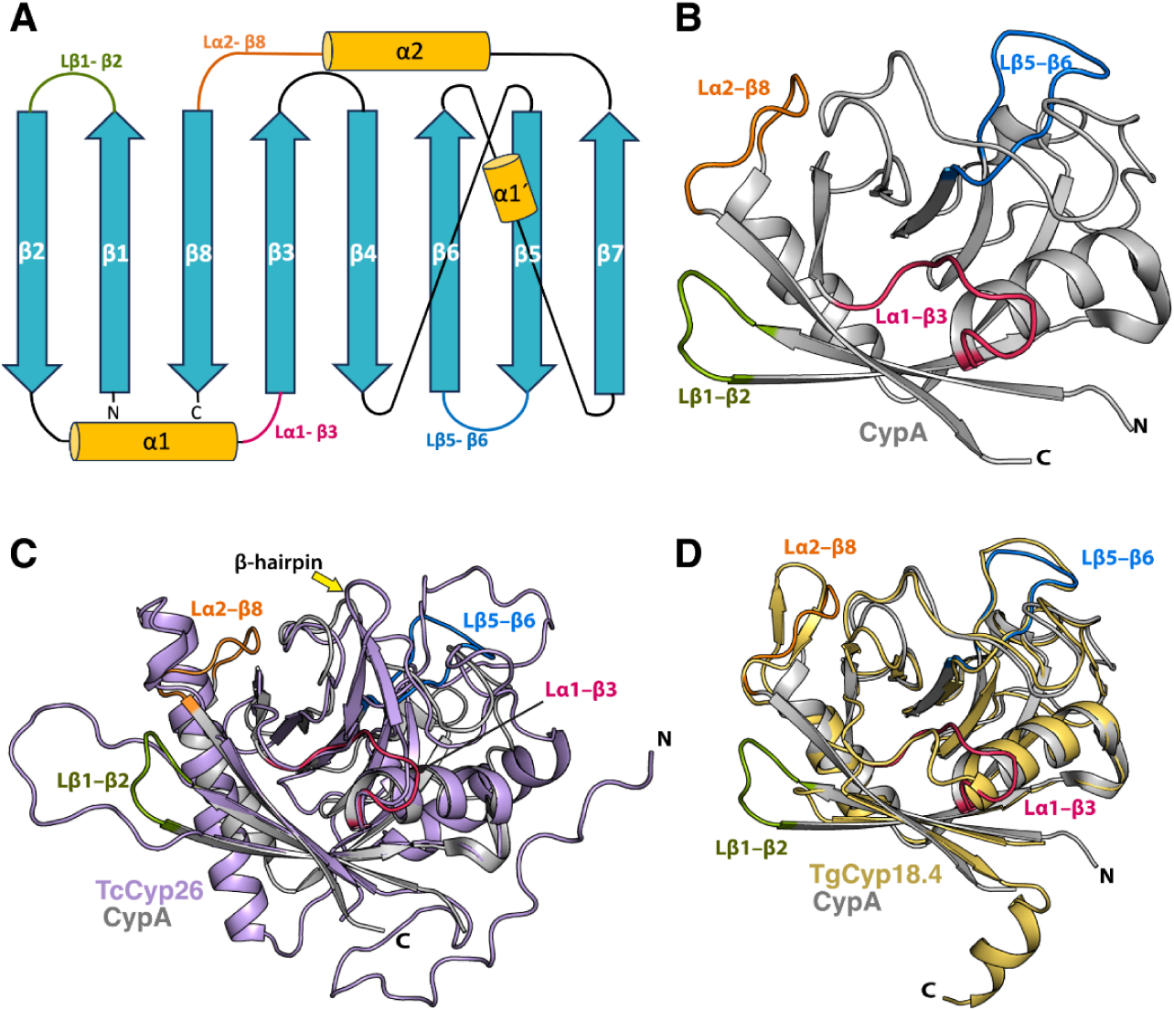
Structural variability in pathogen Cyps. (A) Schematic representation of the topology of human CypA. Loops L_β1−β2_, L_α1−β3_, L_β5−β6_, and L_α2−β8_ are highlighted in green, magenta, blue, and orange, respectively. (B) Three-dimensional structure of human CypA. The protein is shown as a gray cartoon, and loops are highlighted using the same color code as in panel A. (C) Superposition of TcCyp26 AlphaFold (AF) model structure (violet) onto human CypA. A yellow arrow indicates the additional β-hairpin present in TcCyp26, corresponding to an extension of the L_α1−β3_ loop. (D) Superposition of TgCyp18.4 AF model (yellow) onto the human CypA structure. N and C denote the N-terminal and C- terminal regions, respectively.

From an evolutionary perspective, this pattern of structural variability reflects the retention of ancient Cyp subfamilies combined with parasite-specific innovation and lineage-specific loss. Apicomplexans, for instance, lack several host Cyp types but have evolved unique lineages. Their modular domain architecture enables Cyps to integrate into cellular networks as scaffolds or regulators, thereby contributing to functional plasticity and virulence. Profiling this diversity is key for both understanding Cyp evolution and guiding the development of pathogen-specific inhibitors.

## 4 PPIase Activity and Regulation of Pathogen-Derived Cyps

Cyps catalyze the *cis–trans* isomerization of X-Pro peptide bonds, a process that is intrinsically slow and often rate-limiting in protein folding due to the high activation energy required for interconversion between the *cis* and *trans* conformers, despite their small free energy difference.^101^ By stabilizing the twisted transition state, Cyps lower the activation energy and accelerate isomerization by several orders of magnitude (Figure 4). Understanding the detailed kinetic parameters and functional characteristics of Cyps is essential not only for deciphering pathogen-specific protein folding mechanisms and guiding the rational design of selective inhibitors, but also for assessing potential functional redundancy among Cyp family members.

**Figure 4:**
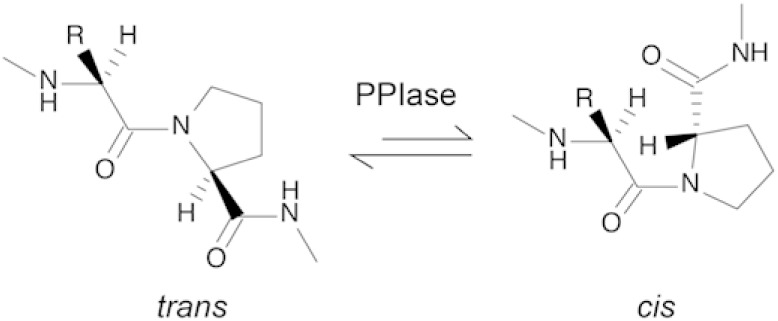
Schematic representation of proline *cis–trans* isomerization. The *cis* and *trans* conformers are represented in the figure, with the *trans* conformer being thermodynamically favored over the *cis* conformer.

Many Cyps from pathogenic organisms have been purified and biochemically characterized (Table 3). The first Gram-negative bacterial Cyp studied was recombinant PpiA (RotA) from *E. coli*.^102^ Despite showing catalytic efficiency comparable to that of human CypA (*k*_cat_/*K*_m_ ≈ 1.3 × 10^7^ M^–1^ s^–1^), it is approximately 1500-fold less sensitive to CsA, with an IC_50_ of 3 μM compared to 2 nM for human CypA, a difference attributed to the substitution of Trp121, a key CsA-binding residue in human CypA, with phenylalanine.

**Table 3:** Catalytic Efficiency and CsA Inhibition of Human and Pathogen-Derived Cyps

Organism	UniProt code	Protein	Catalitic efficiency *k*_cat_/*K*_m_	CsA inhibition	Refs
*H. sapiens*	P62937	CypA	1.46 × 10^7^ M^–1^s^–1^ ^a^		^115^
*H. sapiens*	P62937	CypA	1.6 × 10^7^ M^–1^s^–1^ ^a^	K_i_ = 17 ± 2 nM	^95^
*H. sapiens*	P62937	CypA	4.9 × 10^6^ M^–1^s^–1^ ^a^	IC_50_ = 1 ± 0.3 nM	^88^
*H. sapiens*	Q08752	CypD	2.03 × 10^5^ M^–1^s^–1^ ^a^		^116^
*H. sapiens*	Q9H2H8	CypJ	9.5 × 10^4^ M^–1^s^–1^ ^a^	IC_50_ = 12.1 ± 0.9 μM	^117^
*H. sapiens*	O43447	CypH	1.3 × 10^6^ M^–1^s^–1^ ^a^		^118^
*B. malayi*	Q27450	BmCyp1	7.9 × 10^6^ M^–1^s^–1^ ^a^	IC_50_ = 860 nM	^82^
*S. mansoni*	Q26548	SmCypA	3.6 × 10^5^ M^–1^s^–1^ ^a^	IC_50_ = 72 nM	^119^
*S. mansoni*	Q26548	SmCypA	1.1 × 10^7^ M^–1^s^–1^ ^a^	IC_50_ = 14 ± 4 nM	^83^
*S. mansoni*	Q26551	SmCypB	8.2 × 10^5^ M^–1^s^–1^ ^a^	IC_50_ = 20 nM	^119^
*L. donovani*	Q9U9R3	LdCypA	6.3 × 10^6^ M^–1^s^–1^ ^a^		^120^
*L. major*	O02614	LmCyp19	2.6 × 10^6^ M^–1^s^–1^ ^a^		^23^
*L. major*	O02614	LmCyp19	1.5 × 10^6^ M^–1^s^–1^ ^a^	K_i_ = 5.2 nM	^121^
*P. falciparum*	Q25756	PfCyp19A	1.2 × 10^7^ M^–1^s^–1^ ^a^	K_i_ = 6.9 nM	^122^
*P. falciparum*	Q25756	PfCyp19A	6.3 × 10^6^ M^–1^s^–1^ ^b^	IC_50_ = 10 ± 4.5 nM	^123^
*P. falciparum*	Q8IIK8	PfCyp19B	2.3 × 10^6^ M^–1^s^–1^ ^a^	IC_50_ = 10 nM	^124^
*P. falciparum*	Q8IIK8	PfCyp19B	5.7 × 10^6^ M^–1^s^–1^ ^b^	IC_50_ = 15 ± 3.4 nM	^123^
*T. gondii*	A0A125YL73	TgCyp23	3.8 × 10^6^ M^–1^s^–1^ ^a^	IC_50_ = 0.6 ± 0.1 nM	^88^
*T. gondii*	A0A125YZ79	TgCyp18.4	1.0 × 10^4^ M^–1^s^–1^ ^a^	IC_50_ = 288 ± 25 nM	^88^
*T. gondii*	S8F7V1	TgCyp18	1.4 × 10^7^ M^–1^s^–1^ ^a^	IC_50_ = 32 nM	^125^,^126^
*T. gondii*	A0A125YV51	TgCyp21	(1.9 ± 0.2) × 10^5^ M^–1^ s^–1^ ^a^	IC_50_ = 36 ± 5 nM	^113^
*T. gondii*	S8F5I7	TgCyp38		IC_50_ = 5 nM	^126^
*T. vaginalis*	A2DT06	TvCyp1 (Cyp19)	7.1 × 10^6^ M^–1^s^–1^ ^a^	IC_50_ = 7.5 nM	^39^
*T. vaginalis*	A2DLL4	TvCyp2 (Cyp19.9)	4.5 × 10^6^ M^–1^s^–1^ ^a^	IC_50_ = 5 nM	^40^
*B. pseudomallei*	Q63SS6	BpPpiA (without signal peptide)	(0.5 ± 0.19) × 10^6^ M^–1^ s^–1^ ^c^	K_i_ = 1.8 μM	^127^
			(3.3 ± 0.4) × 10^6^ M^–1^ s^–1^ ^a^		
*B. pseudomallei*	Q63SS5	BpPpiB	(1.3 ± 0.5) × 10^6^ M^–1^ s^–1^ ^c^	K_i_ = 2.4 μM	^127^
			(5 ± 1.69) × 10^6^ M^–1^ s^–1^ ^a^		
*E. coli*	P0AFL3	EcPpiA	1.3 × 10^7^ M^–1^s^–1^ ^a^	IC_50_ = 3 μM	^42^,^102^
*E. coli*	P0AFL3	EcPpiA	5.71 × 10^7^ M^–1^s^–1^ ^a^	IC_50_ = 25–50 μM	^128^
*E. coli*	P0AFL3	EcPpiA	2.0 × 10^7^ M^–1^s^–1^ ^a^	K_i_ = 3.4 ± 0.28 μM	^129^
*E. coli*	P23869	EcPpiB	1.9 × 10^6^ M^–1^s^–1^ ^a^		^130^
*E. coli*	P23869	EcPpiB	6.74 × 10^7^ M^–1^s^–1^ ^a^	IC_50_ = 25–50 μM	^128^
*L. pneumophila*	Q5ZRZ4	LpCyp18(PpiB)	4.6 × 10^6^ M^–1^s^–1^ ^a^	IC_50_ = 1.25 ± 0.1 μM	^131^
*M. tubercolosis*	P9WHW3	MtPpiA	2.0 × 10^6^ M^–1^s^–1^ ^a^		^93^
*S. aureus*	Q7A1C0	SaPpiB	5.0 × 10^6^ M^–1^min^–1^ ^a^		^132^
*S. aureus*	Q7A1C0	SaPpiB	1.2 × 10^5^ M^–1^s^–1^ ^a^		^133^
*S. pneumoniae*	Q8DQG5	SlrA (SpPpiA)	1.1 × 10^6^ M^–1^s^–1^ ^a^	IC_50_ = 880 ± 120 nM	^49^
*S. typhimurium*	P20753	StPpiA	*Vmax* = 0.87 ± 0.14 μmol/min^a^		^134^

a Obtained with AAPF.

b Obtained with ALPF.

c Obtained with AFPF.

Cyps from pathogens exhibit a wide range of catalytic efficiencies, with *k*_cat_/*K*_m_ values ranging from host-like to strongly reduced levels (Table 3). Several Cyp family members show efficiencies ≥10^6^ M^–1^ s^–1^, similar to human CypA. In contrast, others, such as TgCyp18.4, display ∼100-fold lower activity (*k*_cat_/*K*_m_ ∼ 10^4^ M^–1^ s^–1^), primarily due to reduced turnover rates, despite retaining reasonable substrate affinity. Remarkably, such variation often exists within the same organism, as observed between TgCyp23 and TgCyp18.4 in *T. gondii*, suggesting functional specialization among different Cyp family members.^88^

Pathogen-derived Cyps also display varying degrees of sensitivity to CsA (Table 3). Notably, several recombinant Cyps exhibit high susceptibility to CsA, including TvCyp1 and TvCyp2 from *T. vaginalis*, LmCyp19 from *L. major*, PfCyp19A/B from *P. falciparum*, and TgCyp23 from *T. gondii*, all showing IC_50_ values in the nanomolar range (∼0.6–10 nM), similar to those observed for human CypA.^88^ Cyps showing moderate sensitivity include TgCyp18 and TgCyp18.4 from *T. gondii*. In contrast, markedly reduced sensitivity was observed for *E. coli* PpiA and PpiB, *L. pneumophila* LpCyp18, and *S. pneumoniae* Srla (Table 3).

Despite this variation in catalytic efficiency, most Cyps retain relatively broad substrate specificity. Studies employing model tetrapeptides have shown that Cyp catalytic efficiency is only modestly influenced by the identity of the amino acid preceding proline, unlike FKBP-type PPIases, where this position can affect activity by several orders of magnitude.^103^ However, some Cyps exhibit moderate substrate preferences; for instance, enzymes from *Burkholderia* and other pathogens have been reported to convert Ala-Pro motifs more efficiently than Phe-Pro analogs.^49,104,105^ These subtle preferences may reflect coevolution with specific substrate repertoires and could prove useful in identifying physiologically relevant folding partners.

PPIase activity can be further modulated by the redox state of cysteine residues. Increasing evidence indicates that the PPIase activity of Cyps is regulated by redox mechanisms.^83,106–110^ Many microbial Cyps contain conserved cysteine pairs that can form intramolecular disulfide bonds under oxidizing conditions, inducing conformational changes that attenuate or abolish PPIase activity.^83,111^ This reversible thiol-based switch allows Cyps to act as redox sensors: oxidation inactivates the enzyme, while reduction restores activity. For instance, in *Schistosoma mansoni*, SmCypA is inactivated by disulfide bond formation between two cysteines, with full activity restored under reducing conditions. Similarly, *Aspergillus fumigatus* Aspf11, a ∼ 18 kDa secreted allergen, forms a disulfide bond under oxidizing conditions.^59^ This bond may help stabilize the protein in an oxidative environment or function as a redox-sensitive switch, potentially serving as a signaling mechanism in response to oxidative stress, as proposed for *C. elegans* CyP3.^112^ An additional example of redox control is provided by TgCyp21 from *T. gondii*, whose PPIase activity is reduced under oxidizing conditions and restored by reducing agents, including the parasite thioredoxin TgTrx, through a cysteine-dependent thiol–disulfide exchange mechanism involving Cys87 and Cys141.^113^

In some cases, cysteine residues affect Cyp function through mechanisms unrelated to redox regulation. In *Brucella abortus*, PpiB contains a single cysteine (Cys128) required for dimerization.^114^ Mutation to methionine does not impair PPIase activity but prevents dimer formation, significantly reducing intracellular replication and virulence in infection models. This highlights the multifunctional roles of cysteines in Cyp biology, contributing not only to catalytic regulation but also to structural assembly and pathogenesis.

## 5 Cyp Inhibitors

The development of Cyp inhibitors has primarily been driven by their role in human diseases, particularly immunosuppression and viral infections. However, the knowledge gained from these studies is highly applicable to anti-infective drug discovery.

Cyp inhibitors encompass a wide range of structural classes, including cyclic peptides, macrocycles, and small molecules, both natural and synthetic (Figure 5). The prototypical example is CsA, an 11-residue cyclic peptide rich in *N*-methylated residues that binds tightly to the hydrophobic pocket of Cyps. In mammalian cells, the CsA-CypA complex inhibits the phosphatase calcineurin, leading to immunosuppressive effects exploited in transplant medicine. Nonimmunosuppressive Cyp inhibitors derived from the natural product CsA, such as Alisporivir (Debio-025),^135,136^ NIM811,^137,138^ and SCY-635,^139^ have been developed, each containing modifications at positions 3 or 4 of the CsA scaffold (Figure 5A).

**Figure 5:**
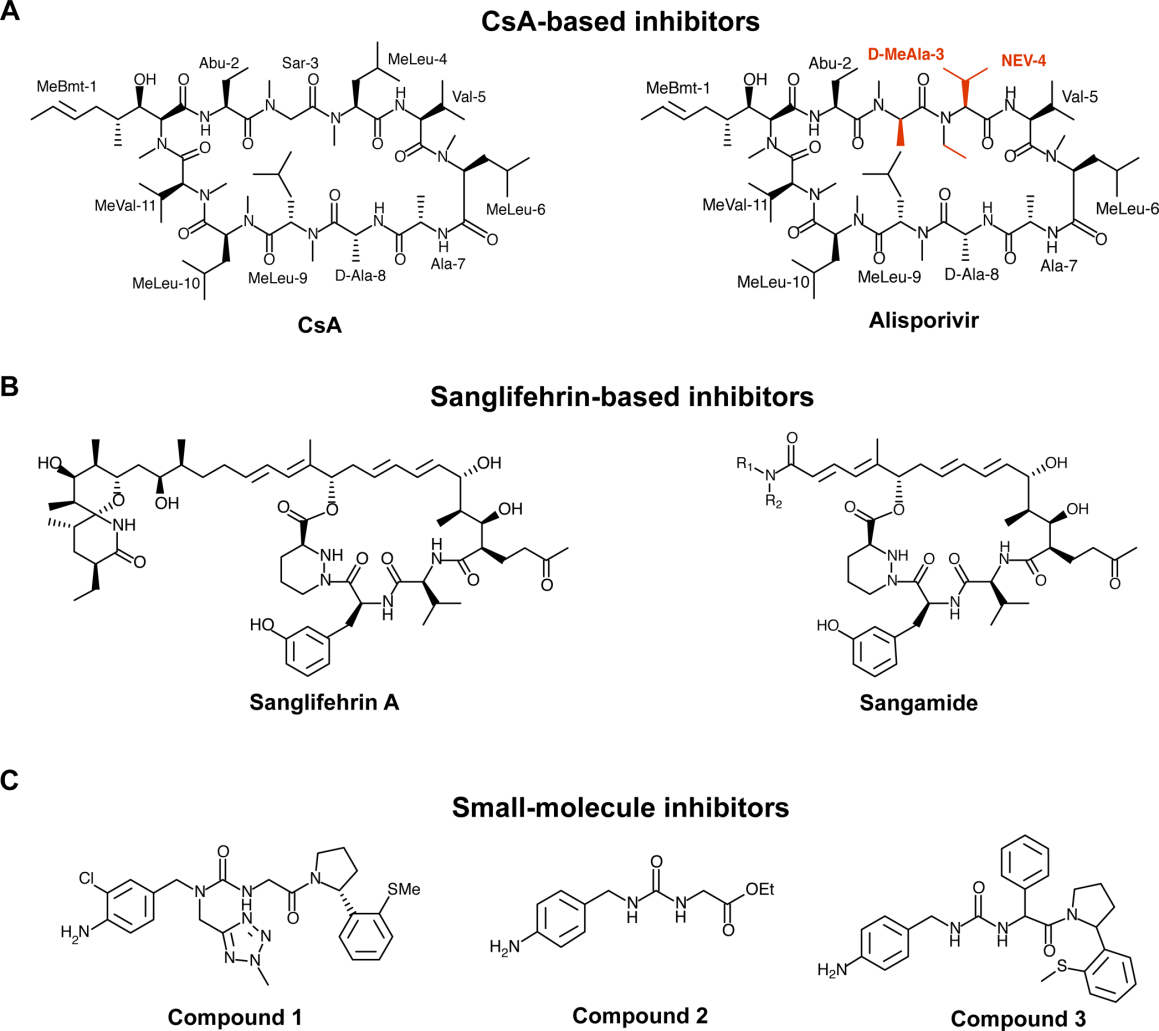
Major classes of Cyp inhibitors. (A) The cyclic peptide CsA and its nonimmunosuppressive analogue Alisporivir that carries substitution in position 3 and 4 (indicated in red on the molecule backbone). (B) Sanglifehrin and its semisynthetic amide derivative Sangamide. (C) Synthetic small-molecule inhibitors. Coumpound 1 is a trivector inhibitor of human CypB,^147^ while compounds 2 and 3 are urea derivatives.^71,145^

Interestingly, CsA and its analogues also exhibit potent antiparasitic effects across multiple species. However, while originally attributed to calcineurin inhibition, this mechanism remains uncertain in many parasites, since calcineurin essentiality has not been clearly established. Evidence from *P. falciparum* and *T. gondii* suggests that CsA activity does not always correlate with PPIase inhibition or calcineurin targeting. Some analogs, such as valspodar, inhibit parasite growth despite weak Cyp binding, suggesting alternative targets such as P-glycoprotein disruption.^140^ Nonetheless, recent studies have renewed interest in Cyps as primary targets. CsA-resistant *P. falciparum* mutants often carry mutations in Cyp genes (PfCyp19A/B) or calcineurin subunits, and recombinant Cyp-CsA complexes can inhibit parasite calcineurin activity.^38^ In other parasites, such as *Leishmania* and *C. elegans*, low sensitivity to CsA may reflect Cyp redundancy or poor drug uptake. Notably, nonimmunosuppressive analogs like [MeVal^4^]-CsA retain strong Cyp binding but minimal calcineurin inhibition, reinforcing the value of targeting Cyps independently of calcineurin.

Beyond CsA, the sanglifehrin family, a group of large macrocyclic polyketides from *Streptomyces*, represents another class of potent Cyp inhibitors (Figure 5B).^141^ Semisynthetic derivatives such as sangamides^142^ have been optimized for pharmacokinetics and reduced toxicity, showing nanomolar efficacy against HCV and synergy with direct-acting antivirals, while avoiding inhibition of multidrug resistance transporters (Figure 5B).

CsA- and sanglifehrin-based inhibitors remain the only Cyp-targeting compounds to have reached clinical trials. However, despite their potency, they are pan-selective and exhibit poor drug-like properties, such as high molecular weight, low solubility, and limited bioavailability, posing significant challenges for further development. These limitations have driven the search for more druggable, selective Cyp inhibitors. Small-molecule Cyp inhibitors have shown promise, though achieving selectivity is difficult due to the high conservation of the active site and surrounding binding pockets (Figure 5C).^143^ Some progress has been made in this area. Selective inhibition of human CypA over human CypB has been achieved with arylindanyl ketones.^144^ Peterson et al. developed potent human CypD inhibitors using biphenyl dicarboxylate macrocycles, though cell delivery required a prodrug strategy and pharmacokinetic data were lacking.^70^ Other chemical series, such as phenyl-pyrrolidine ureas, demonstrated strong inhibition across multiple isoforms but lacked specificity.^145^ Later refinements of these scaffolds yielded improved CypD inhibitors, but comprehensive data on selectivity, pharmacokinetics, and safety remain limited.^71,146^ More recently, optimization of trivector CypB-targeted inhibitors, extending into the “three o’ clock” pocket, has produced a low-genotoxicity compound with a K_d_ of 12 nM for human CypB and >9- and >12-fold selectivity over CypA and CypD, respectively, demonstrating promising antifibrotic activity in MASH-relevant cellular models.^147^

Remarkably, recent advances in Cyp targeting also include the development of PROTACs (Proteolysis Targeting Chimeras) based on CsA and Sanglifehrin scaffolds. These bifunctional molecules enable targeted degradation of Cyps, offering an alternative therapeutic strategy beyond enzymatic inhibition.^148,149^ To date, such PROTACs have been developed against human Cyps and have been shown to promote efficient target degradation while dissociating Cyp engagement from calcineurin inhibition, thereby avoiding immunosuppressive effects. This proof of principle suggests that PROTAC-based approaches could potentially be extended to selectively target pathogen-derived Cyps.

Further research into nonimmunosuppressive CsA derivatives is essential. Structural analysis of the ternary human complex CypA-CsA-calcineurin reveals how CsA binds to calcineurin and provides key insights into potential chemical modifications that could guide the synthesis of such derivatives (Figure 6).

**Figure 6:**
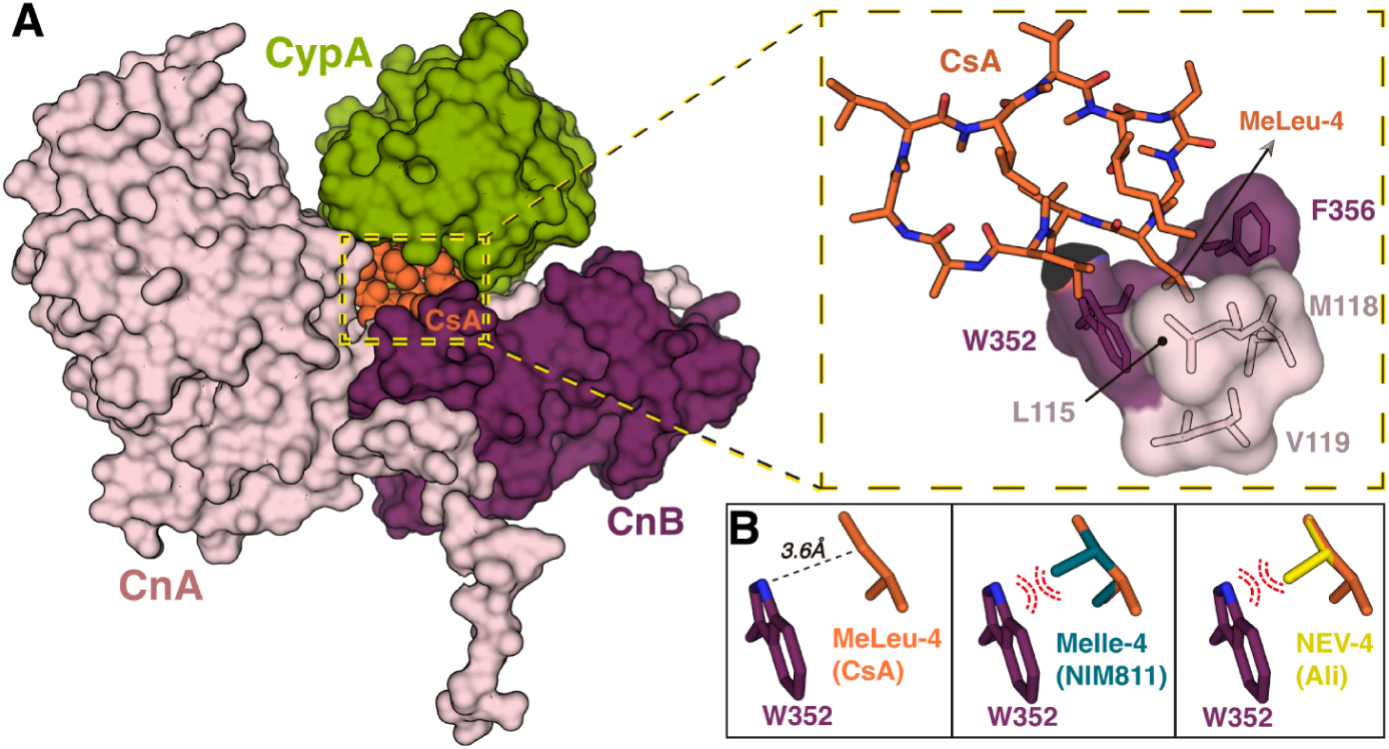
CsA interaction with calcineurin (Cn). (A) Ternary CypA-CsA-Cn complex (PDB: 1M63). CypA and Cn (CnA and CnB) are depicted as surfaces, with CypA in green, CnA in pink, and CnB in purple. CsA is represented as orange spheres. On the right, a zoomed view of the CsA interface shows the hydrophobic cavity where MeLeu-4 of CsA is placed. CsA is drawn in sticks, and the Cn residues involved in MeLeu-4 stabilization are displayed as transparent surface. (B) Predicted spatial accommodation of residue 4 in CsA analogs. In the left box, the distance between MeLeu-4 of CsA and Trp352 from Cn is indicated. In the middle and right boxes, the residues MeIle-4 (from NIM811) and *N*-ethyl-valine-4 (from Alisporivir) are superposed onto the MeLeu-4 of CsA. Steric hindrance and predicted atomic clashes between the additional methyl groups introduced by CsA modifications and Trp352 are highlighted as red dashed lines. Trp352, CsA, NIM811, and Alisporivir are shown in purple, orange, blue, and yellow sticks, respectively.

The structural characterization of the ternary complex enables a detailed exploration of the molecular interactions between CsA and calcineurin (Figure 6A). CsA establishes both polar and hydrophobic interactions with CypA and calcineurin. Notably, the residue MeLeu-4 of CsA plays a critical role in the inhibition of calcineurin. Its side chain is tightly accommodated within a well-defined hydrophobic pocket formed by Leu115, Met118, Val119, Trp352, and Phe356 of the calcineurin protein (Figure 6A, zoomed view). Any structural modification at this position is likely to result in steric clashes with the aforementioned residues. For example, the CsA derivatives NIM811 and Alisporivir contain substitutions at residue 4 with Me-Ile and *N*-ethylvaline, respectively. The additional methyl group introduced by these side chains is incompatible with the spatial constraints of the hydrophobic pocket, particularly due to potential clashes with Trp352 (Figure 6B). This structural incompatibility would prevent the formation of the ternary complex.

In summary, these CsA analogs with substitutions at residue 4 retain high CypA-binding affinity but lack the calcineurin interaction motif, thus avoiding immunosuppression while maintaining strong antiviral activity, especially against hepatitis C virus. Structural data support their binding modes, and multiple crystal structures with Cyps have been reported (Table 1).

## 6 Challenges and Perspectives

Despite their recognized importance as critical contributors to virulence, immune evasion, and antimicrobial resistance, major questions remain regarding the precise mechanisms of action and potential druggability of pathogen-derived Cyps. Functionally, many Cyp-dependent processes are still only partially understood. For example, how Cyps modulate secretion systems or signaling pathways, and which proteins they interact with, remain open questions. Addressing these issues will require systematic mapping of Cyp interaction networks and identification of their substrates in pathogenic organisms.

An additional unresolved issue concerns functional redundancy. Parasites such as *T. cruzi* and *T. gondii* encode a large number of Cyp family members, raising the question of whether these proteins serve distinct roles or are functionally interchangeable. Emerging evidence supports the former; many Cyps appear to carry out specialized, nonredundant functions, pointing to a high degree of functional diversification.

From a therapeutic standpoint, targeting Cyps is appealing given their importance, but remains challenging due to their widespread presence in the host. To date, Cyp inhibitors that have entered clinical development are largely pan-selective. Indeed, structural studies reveal that drug-binding pockets in Cyps are often conserved across species, raising both an opportunity and a concern: broad-spectrum Cyp inhibitors might combat multiple infections, yet off-target effects on human Cyps could cause toxicity. Encouragingly, recent advances have demonstrated the feasibility of developing more selective inhibitors.

Looking ahead, future drug discovery efforts will likely focus on exploiting subtle structural differences between host and pathogen Cyps, identifying less conserved allosteric sites suitable for selective inhibition, and exploring innovative therapeutic strategies, including PROTAC-based approaches to induce selective degradation of pathogen Cyps. Together, these strategies hold promise for overcoming current limitations and unlocking the therapeutic potential of this protein family.

## Supplementary Material



## Notes

Table S1: structural differences between *T. gondii* cyclophilins and human CypA; Table S2: structural differences between *T. cruzi* cyclophilins and human CypA; Figure S1: structural variability in pathogen cyclophilins (PDF)
