# Supplementary material for: Structural and Kinetic Variability of Pathogen Cyclophilins: Functional Diversification and Therapeutic Potential
Source: ACS Infect Dis. 2026 Apr 22;12(5):1538–54. doi: 10.1021/acsinfecdis.6c00016 (PMC13162259; doi:10.1021/acsinfecdis.6c00016)
Supplement: Supplementary file 1 [file id6c00016_si_001.pdf]

SUPPORTING INFORMATION

Structural and kinetic variability of pathogen cyclophilins: functional diversification and therapeutic potential

Filippo Favretto [a, #], Eva Jiménez-Faraco [b, #], Silvia Fruncillo [a], Nicola Masè [a], Paola Dominici [a], Juan A. Hermoso [b, *], Alessandra Astegno [a, *]

[a] *Department of Biotechnology, University of Verona, Strada Le Grazie 15, 37134 Verona, Italy.*
[b] *Department of Crystallography and Structural Biology, Institute of Physical Chemistry Blas Cabrera (IQF), CSIC, Serrano 119, 28006 Madrid, Spain.*

[#] These authors contributed equally.
*Corresponding authors. Alessandra Astegno, e-mail: alessandra.astegno@univr.it; Juan A. Hermoso, e-mail: xjuan@iqf.csic.es

Table S1. **Structural differences between *T. gondii* cyclophilins and human CypA**.

Protein	Uniprot code	N-terminus	Loop $_{\beta1-\beta2}$	Loop $_{\alpha1-\beta3}$	Loop $_{\beta5-\beta6}$	Loop $_{\alpha2-\beta8}$	C-terminus
TgCyp18	S8F7V1	+		-			
TgCyp18.4	A0A125YZ79		-	-		+	+
TgCyp21	A0A125YV51	+	-	-			
TgCyp23	A0A125YL73	+		+			
TgCyp26	A0A125YLU4	+		+			
TgCyp32	S8FB56	+		+			+
TgCyp35	A0A125YQ35	+		+			+
TgCyp38	S8F5I7	+		+			
TgFCB57	S8F548	+		+		+	+
TgCyp64	A0A125YVH7	+	-	-	+	+	+
TgCyp66.21	A0A125YII8			+		+	+
TgCyp66.25	S8GFQ1	-	-	-	+	+	+
TgCyp69	A0A125YUW2	+	-	-		+	+
TgCyp86	S8FD30	+	-	-		+	

The analysis has been done by the superposition of AlphaFold models or X-ray structures deposited in PDB.
Protein identifiers have been designated according to the molecular weight of each protein.
[+] indicates a larger range of residues compared to human CypA.
[-] indicates a shorter range of residues compared to human CypA.

Table S2. **Structural differences between *T. cruzi* cyclophilins and human CypA**.

Protein	Uniprot code	N-terminus	Loop β1- β2	Loop α1- β3	Loop β5- β6	Loop α2- β8	C-terminus
TcCyp19 (TcCypA)	Q4E4L9	+					
TcCypB	Q4DPB9	+				+	
TcCyp20	Q4DC03		-	-	+	+	+
TcCyp21	Q4DNC9	+				+	
TcCyp22	Q4DI85	+		+		+	+
TcCyp24	Q4CXV1 / Q4D7C3	+	+	+			
TcCyp25	Q4DFL3	+		+			+
TcCyp26.2	Q4DJE5	+		+		+	+
TcCyp28	Q4CX88	+		+			+
TcCyp26	Q4DU72	+	+	+	+	+	
TcCyp30	Q4DNS3	+		+		+	+
TcCyp35	Q4DM35	+		+		+	+
TcCyp29	Q4DQI8	+		+		+	+
TcCyp40	Q4E4G0			+		+	+
TcCyp103	Q4D1M5	+	-	-		+	+

The analysis has been done by the superposition of AlphaFold models or X-ray structures deposited in PDB.

$^{+}$ indicates a larger range of residues compared to human CypA.

$^{-}$ indicates a shorter range of residues compared to human CypA

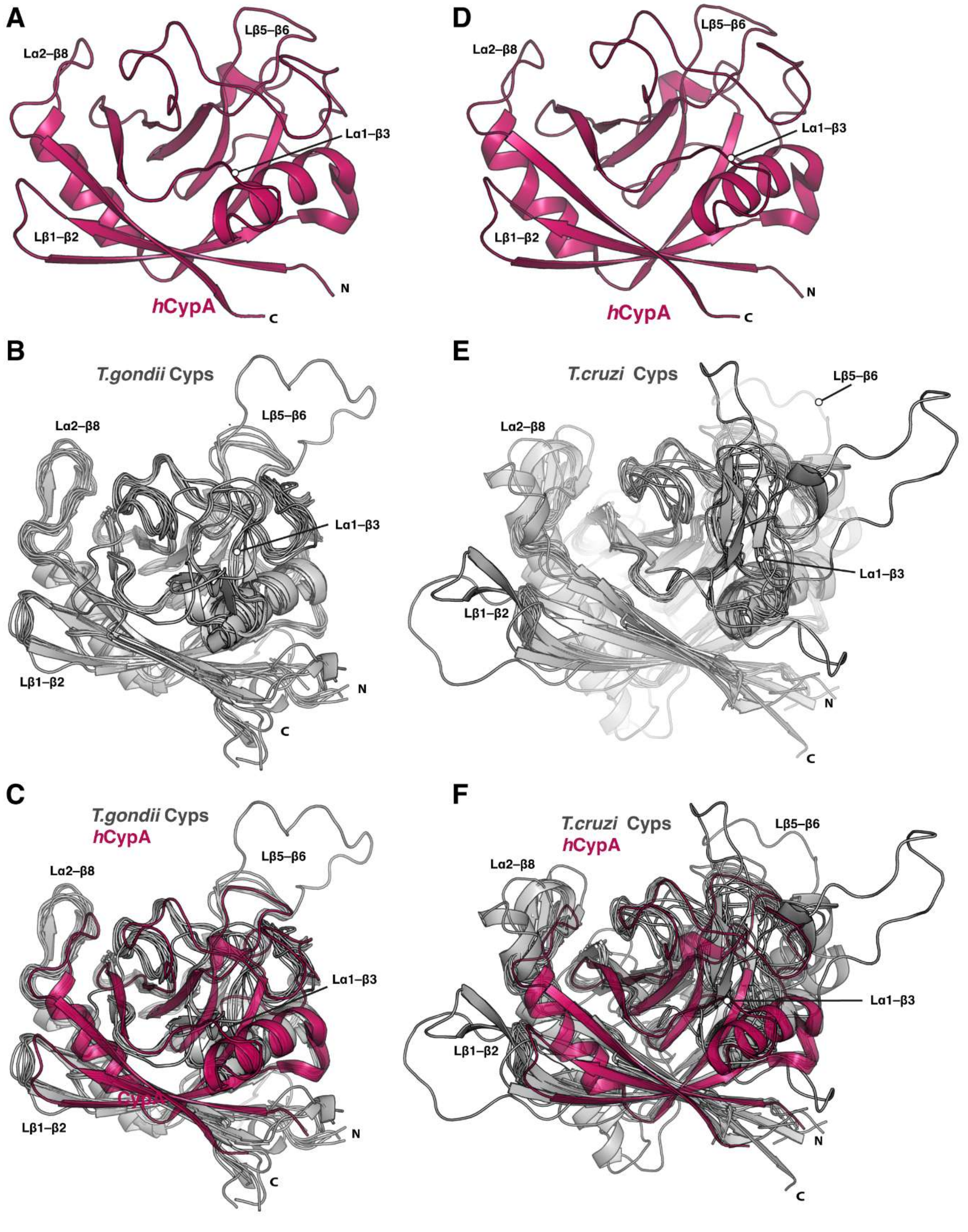

Figure S1. **Structural variability in pathogen cyclophilins**. **(A)** Three-dimensional structure of human CypA. The protein is shown as magenta cartoon. Loops $L_{\beta1-\beta2}$, $L_{\alpha1-\beta3}$, $L_{\beta5-\beta6}$ and $L_{\alpha2-\beta8}$ are labeled; N and C denote the N-terminal and C-terminal regions, respectively. (**B)** Sequence alignment of *T. gondii* cyclophilins. **(C)** Superimposition of hCypA (magenta) onto the *T. gondii* cyclophilins. **(D)** hCypA three-dimensional structure. (**E)** Alignment of all *T. cruzi* cyclophilins. (**F)** Superimposition of hCypA (magenta) onto the *T. cruzi* cyclophilins.
